# Of Elephants and Other Mammals: A Comparative Review of Reproductive Tumors and Potential Impact on Conservation

**DOI:** 10.3390/ani12152005

**Published:** 2022-08-08

**Authors:** Lisa M. Abegglen, Tara M. Harrison, Anneke Moresco, Jared S. Fowles, Brigid V. Troan, Wendy K. Kiso, Dennis Schmitt, Amy M. Boddy, Joshua D. Schiffman

**Affiliations:** 1 Department of Pediatrics & Huntsman Cancer Institute, University of Utah, Salt Lake City, UT 84112, USA; 2 Arizona Cancer Evolution Center, Arizona State University, Tempe, AZ 85281, USA; 3 Exotic Species Cancer Research Alliance, North Carolina State University College of Veterinary Medicine, Raleigh, NC 27607, USA; 4 Department of Clinical Sciences, North Carolina State University College of Veterinary Medicine, Raleigh, NC 27607, USA; 5 Reproductive Health Surveillance Program, Morrison, CO 80465, USA; 6 White Oak Conservation Foundation, Yulee, FL 32097, USA; 7 Department of Animal Science, William H. Darr College of Agriculture, Missouri State University, Springfield, MO 65809, USA; 8 Department of Anthropology, University of California Santa Barbara, Santa Barbara, CA 93106, USA; 9 Peel Therapeutics, Inc., Salt Lake City, UT 84108, USA

**Keywords:** *Elephas maximus*, *Loxodonta africana*, fertility, leiomyoma, neoplasia, prevention, treatment, uterine, fibroid, comparative oncology

## Abstract

**Simple Summary:**

Both Asian and African elephants are endangered, and conservation efforts aim to minimize negative impacts to each species, while increasing their population sizes. Understanding factors that impact reproduction are important for conservation. Tumors can negatively impact reproductive success, particularly when they are located in the reproductive system. This article reviews the prevalence of reproductive tumors in elephants and other mammals. The impact of human tumors and treatment options are also reviewed as a comparative approach to consider potential treatment approaches for elephants diagnosed with reproductive tumors. Future studies are needed to understand the impact of these tumors on elephant conservation and to establish fertility preserving treatments.

**Abstract:**

Reproductive tumors can impact conception, pregnancy, and birth in mammals. These impacts are well documented in humans, while data in other mammals are limited. An urgent need exists to understand the reproductive impact of these lesions in endangered species, because some endangered species have a documented high prevalence of reproductive tumors. This article documents that the prevalence of both benign and malignant neoplasia differs between African and Asian elephants, with Asian elephants more frequently diagnosed and negatively affected by both. The prevalence of these tumors across mammalian species is compared, and impact plus treatment options in human medicine are reviewed to inform decision making in elephants. Evidence suggests that reproductive tumors can negatively impact elephant conservation. Future studies that document reproductive outcomes, including the success of various treatment approaches in elephants with tumors will benefit conservation efforts.

## 1. Introduction

All species of elephants (*Elephas maximus*, *Loxodonta africana*, and *Loxodonta cyclotis*) are endangered due to poaching, habitat loss, disease susceptibility (including elephant endotheliotropic herpesvirus and other infectious diseases), and additional threats related to climate change [1,2,3]. Ongoing in situ and ex situ conservation efforts aim to increase population size, maintain genetic diversity, and protect these animals that play a crucial role in our ecosystems [4,5]. These large-bodied land mammals are able to clear trees in forests, create water holes, and fertilize soil, which enables smaller species of animals and plants to thrive [6]. However, their title as the largest land mammals also means that conservation strategies focused on elephant reproduction face unique challenges not faced in other species. In addition to ex situ breeding difficulties related to housing and assisted reproduction due to their tremendous size, elephants have the longest estrous cycle (13–17 weeks) and gestation period (20–23 months) of any mammal [7]. As a comparison, humpback whales and orcas have an average gestation of 12 months [8] and 18 months [9], respectively. In addition to the relatively long gestation, elephants typically give birth to only one calf and have a long dependency period with each calf weaning between two to five years old. While elephant lifespans can be long (70+ years) [10,11], the total average reproductive output over their lifetimes is relatively low (4–5 offspring) compared to other long-lived species, such as whales. This slow reproductive strategy is magnified by the long intervals between pregnancies, which are influenced by long lactation periods of approximately three years. The interbirth interval is approximately five years, but occasionally as long as 10 years in free-ranging African and Asian elephants [11,12,13,14]. Lastly, social networks also contribute to elephant reproduction and neonatal survival. The presence of grandmother and mother are important for calf survival and daughter’s reproduction [11]. Considering that every elephant pregnancy and successful birth contributes to conservation efforts, all factors that may negatively impact reproductive success should be considered and strategies to counteract them should be in place.

Unfortunately, elephants are vulnerable to abnormal growths in reproductive tissues, which can impact reproductive success in these endangered species. Asian elephants in particular are often diagnosed with benign uterine tumors called leiomyomas [15,16] or fibroids (the term typically used to describe these lesions in humans) [17]. While malignant tumors overall are rare in elephants, when they do occur, the reproductive tract is disproportionately affected [15,16]. Benign and malignant reproductive tract tumors are known to affect reproduction and pregnancy in other animals [18,19,20,21], and even tumors outside of the reproductive tract can have significant negative impacts on reproductive success [21,22]. This article takes a comparative approach to summarize reproductive tumors across species in an attempt to better understand the significance of these tumors in elephants. First, reported reproductive neoplasias in elephants are summarized. Followed by a review of the prevalence and impact of uterine leiomyomas in other mammals. This comparative perspective can identify the most vulnerable species, highlight the large variation in prevalence across species, and thereby reveal species that may have evolved novel mechanisms of defense to prevent these lesions. Additionally, because human tumors are the most well studied and characterized of all species, the impacts of tumors, including uterine leiomyomas, on human reproduction are summarized. Approaches to preserve fertility in humans with tumors are discussed to assess potential opportunities to preserve fertility in elephants negatively impacted by tumors. Lastly, potential risk factors for uterine leiomyoma and their impact on reproductive success in elephants are discussed, as well as potential treatment approaches that could be applied not only to uterine leiomyoma, but also to other types of cancer in elephants.

## 2. Comparative Reproductive Oncology Can Provide New Insights into Treatment

Reviewing comparative reproductive oncology offers a means to study risk factors [23] as well as identify potential treatments for neoplasia across multiple species [24]. Learning more about risk factors of reproductive tumors and how to lower the probability of developing these tumors is especially important in endangered species where reproduction remains key to breeding programs and conservation. Importantly, even if a reproductive tumor is diagnosed early and the animal survives, that animal’s reproductive potential may still be compromised. In addition to animal health, comparative reproductive oncology may also advance human health, and in fact, both comparative oncology and reproductive disorders are part of the One Health approach [24,25]. As environmental changes, habitat loss, and poaching continue to threaten all animal species, large and long-lived species, such as elephants, are disproportionately vulnerable, and the interconnectedness of animal health, planetary health, and fertility becomes more apparent [26].

## 3. Prevalence of Reproductive Tumors in Elephants

Neoplasia, a term commonly used in veterinary medicine, is defined as the presence or formation of a new, abnormal growth of tissue [27]. A tumor is a mass or swelling caused by abnormal cell growth [28], which is either benign or malignant. Benign tumors do not invade surrounding tissues or metastasize (spread) to other parts of the body, while malignant tumors (cancer) often do [29]. A lesion is an abnormal change in organ or tissue structure caused by injury or disease [30]. A tumor is a type of lesion, but not all lesions are tumors. As elephants age, both Asian and African elephant females exhibit reproductive lesions that can impair conception, implantation, pregnancy or labor. Although abnormal lesions in the reproductive tract are common in both Asian and African elephant species, uterine leiomyomas are more common in Asian elephants [7], [31] and are more prevalent and larger in older, nulliparous females [32]. While some cases can present with an intermittent bloody discharge, the majority of cases do not exhibit overt clinical signs and do not generally result in death. 

The prevalence of uterine leiomyoma in Asian elephants reported by several studies is 30–100% [15,16,32,33,34,35,36,37,38,39] (Table 1). In contrast, uterine leiomyoma rarely occurs in African elephants, which have only been reported to develop vestibular polyps with a prevalence of 70% in females over 30 years of age [7]. Other reproductive tract neoplasias have been identified in elephants as well, but are less common. In a recent archival review of 80 adult female Asian elephant mortalities in the United States from 1988 to 2019, neoplasms were identified in 64 cases (80%). Besides the high prevalence of typically benign uterine leiomyomas at 71% (57/80), seven other malignant neoplasias were identified in 17.5% of female elephants (14/80), including eight uterine adenocarcinomas [16] (Table 1). Other case reports identified uterine adenocarcinoma, bilateral multilocular serous ovarian cystadenoma, and an undifferentiated malignant neoplasm of the uterus in Asian elephants [15,40,41] (Table 1). Non-neoplastic proliferative lesions in multiple areas of the reproductive tract that affect both African and Asian elephants alike have been reported to occur in the form of cysts, polyps, and hyperplastic endometrial disease [7,15,16,31,33] (Table 1). Although the combined prevalence of malignant reproductive tumors in elephants is estimated at under 6% of summarized study cases, benign lesions have the potential to result in impaired reproductive success.

## 4. Uterine Leiomyoma as a Case Study to Understand Potential Reproductive Consequences of Tumors in Veterinary Patients

Leiomyomas have been reported to occur in a wide range of species: human, bongo, rhinoceros, felids, canids, primates, and elephants, among others [16,32,42,43,44,45]. The literature on neoplasia often does not include the unaffected population, making it difficult to estimate prevalence, but studies that do report these benign lesions show a wide variation in leiomyoma prevalence across mammalian species (Table 2 and Table 3). These species-specific differences in prevalence suggest a genetic component to these benign lesions, where some species may have evolved better defenses against uterine leiomyomas than others. Similar to observations in other species, uterine leiomyomas in elephants can negatively affect reproduction without causing death. While reported prevalences vary among humans and great apes, comparisons are hampered by differences in methods of data collection across studies. Accurately assessing incidence and prevalence of leiomyomas in women is challenging, because unlike animals cared for in American Zoological Association (AZA) accredited institutions, most women are not examined for tumors at death. A systematic review of published studies reporting uterine leiomyoma prevalence in women between 1995 and 2015 found that prevalences ranged from 4.5% to 68.6% [46]. This broad range results from differences in study populations and methodologies. Because differences in study methodologies also exist between human and animal studies, comparing prevalence across primates or between humans and other primates is challenging. In addition, the presence and prevalence of these leiomyomas in the free-ranging counterparts of many of these non-human species is unknown since necropsies of free-ranging populations are rare, and often, sample quality is inadequate due to autolysis, and a thorough examination for uterine pathologies may not be performed. However, prevalence across non-human animals can be compared with more consistent study protocols, and differences in prevalence are seen at higher taxonomic levels, as Table 2 shows: felids are more prone to leiomyomas than canids and suids more so than tayassuidae. Some ungulates, such as the bongo, seem to be particularly prone to developing leiomyomas [42].

Risk factors for uterine leiomyomas are not completely elucidated, and this is an active field of investigation in both human and veterinary medicine [44,47]. Both genetic and life factors likely contribute to leiomyoma risk, as, even within the same species, differences in disease prevalence are observed between populations. Reproductive history is a shared risk factor associated with leiomyoma development across species. For example, nulliparity is an established risk factor across many species [16,32,42,43,44,45,47,48]. Exposure to endogenous hormones without pregnancy, specifically estrogen, is positively correlated with the risk of uterine leiomyomas in humans [49,50]. Known human risk factors, such as high gonadotropin levels, should be explored in veterinary patients for their influence on uterine leiomyoma development across species.

Among humans, multiple studies report genetic factors associated with the development of uterine leiomyoma [51,52,53], suggesting that genetic predisposition plays a role. Animals likely also have some unidentified genetic predispositions that contribute to disease risks, as genetic differences between species and within species can influence risk. Within similarly managed populations, marked differences can be observed between related species; for example, a very high prevalence of leiomyomas in Asian elephants (30–100%) versus a very low prevalence in African elephants (0%) (Table 2). In addition to genetic factors, indirect factors may be associated with this difference, such as the higher percentage of acyclic African elephant females compared to Asian elephants in managed populations [54], and where acyclicity may reduce leiomyoma growth [55]. Leiomyomas are also commonly reported in chimpanzees, and differences in prevalence between laboratory (53.6%) and sanctuary (8.8%) chimpanzees have been observed. These differences may be due to differences in reproductive management (separating the sexes and/or use of contraceptives) of females, how often they are pregnant (as opposed to constant hormone cyclicity when not pregnant), or due to differences in tumor diagnosis approach (necropsy vs. ultrasound).

The Exotic Species Cancer Research Alliance (ESCRA) is a neoplasia database established to collect and record neoplasia cases in non-domestic species across facilities, including those not reported in the literature [56,57]. Cases of neoplasia and corresponding treatments are collected from multiple zoological and aquatic institutions, as well as veterinary teaching hospitals and private practices. These cases are continually collected to determine which species develop neoplasia, if and how they are treated, and their outcomes (survival and adverse effects). This supplements typical reports in the literature, which often consist of interesting case reports, case series, or cases from a single institution or laboratory evaluating prevalence only [58,59]. Table 3 reports the prevalence of leiomyomas out of total cases of neoplasia reported to ESCRA. This list includes a leiomyoma in an African lion that was surgically removed by ovariohysterectomy, and a leiomyoma in a chimpanzee that was inoperable due to its location within the pelvic canal (T.M. Harrison, unpublished data). Both cases (chimpanzee and lion) were diagnosed when the animals were near the end of their reproductive lifespan and reproductive outcomes prior to diagnosis were not documented. Therefore, the impact on their reproduction cannot be evaluated. Similar to cases in the literature, most of the cases documented in ESCRA were either diagnosed at the time of death or not treated. Uterine leiomyomas can be identified in living animals by ultrasound, but these examinations are only performed when medically necessary. Limited diagnosis in living animals means that most animals do not receive treatment for these lesions. However, examples of fertility preserving treatments in domestic animals do exist. Surgery was performed to remove a uterine leiomyoma in a Holstein cow [19], and post-surgery, the animal successfully became pregnant and gave birth. In another example, a horse with a leiomyoma in the right uterine horn underwent a partial ovariohysterectomy and reproductive potential was maintained [18]. Although both of these cases were diagnosed and treated successfully, early diagnosis and successful treatment of tumors is uncommon in non-domestic animals. While fertility preservation treatments are rarely attempted in non-domestic animals diagnosed with tumors, gamete rescue has been performed in other situations. Successful birth of live animals using this approach varies between species [60,61,62,63].

In addition to elucidating the risk factors that contribute to the development of leiomyomas and potential treatment approaches, it is necessary to investigate the repercussions of these benign tumors. Uterine leiomyomas have the potential to hamper pregnancy, but a lack of pregnancy is a risk factor for leiomyomas. Therefore, it is difficult to determine which issue (lack of pregnancy or leiomyoma) develops first without regular reproductive exams early in life, before leiomyomas develop.

Reports of negative impacts of these tumors in non-domestic animals exist [39,64,65,66]. For example, in the endangered greater one-horned rhinoceros (*Rhinoceros unicornis*) reproductive tract tumors grew large enough to cause infertility by the age of 18, out of a typical reproductive lifespan of 28 years, and overall lifespan of 40 years [64]. These animals were diagnosed and evaluated through ultrasound examination, and 100% of screened rhinos over 12 years of age had presumed leiomyomas present in the cervix, vagina and uterus, and 72.2% of rhinoceros with reproductive tract tumors were not successful breeders. Furthermore, 33% of these animals were presumed to be infertile due to the size or number of these tumors. The presence of tumors and reduced fertility was more common in animals bred later in life compared to those bred successfully earlier in their life. This example highlights the need to establish preventative and treatment approaches to decrease the impact of uterine leiomyomas in affected species. 

**Table 2:** Summary of studies evaluating prevalence of uterine leiomyomas, total populations included individuals without neoplasia.

Species	Uterine Leiomyoma Cases/Total Population Assessed (Prevalence)	Population Assessed	Reference
Primates			
Human (*Homo sapiens*)	* (4.5–68.6%)	Europe	[67,68,69,70]
		North America	[71,72,73]
		South America	[74]
		Africa	[75,76,77,78]
		Asia	[79,80,81,82]
		Middle East	[83]
Chimpanzee (*Pan troglodytes*)	9/16 (56.3%)	Laboratory, >35yr	[84]
	20/32 (62.5%)	Laboratory, >35y	[85]
	7/80 (8.8%)	African Sanctuaries	(Moresco and Feltrer unpublished data)
Gorilla, Mountain gorilla (*Gorilla beringei beringei*) Gorilla, western lowland (*Gorilla gorilla gorilla*)	Not reported (0%)		[86]
	3/14 (21%)	U.S. zoos	[87]
Orangutan (*Pongo* spp.)	3/24 (12.5%)	U.S. zoos	[88]
Proboscidea (elephants)			
Asian elephant (*Elephas maximus*)	57/80 (71.3%)	U.S. zoos	[16] **
	27/27 (100%)	U.S. and European zoos	[32] ***
		U.S. zoos	
	19/27 (70.3%)	U.S. zoos	[33]
African elephant (*Loxodonta africana*)	0/8 (0%)	U.S. zoos	[32]
	0/13 (0%)	U.S. zoos	[33]
Perissodactyla (odd-toed ungulates)			
Rhinoceros, greater one-horned (*Rhinoceros unicornis*)	4/5 (80%)	U.S. and European zoos	[32]
Artiodactyla (even-toed ungulates)			
Bongo (*Tragelaphus eurycerus*)	9/11 (81.8%)	U.S. zoos	[42]
Suidae	32/97 (33%)	U.S. zoos	[89]
Tayassuidae	0/11 (0%)	U.S. zoos	[89]
Carnivora (carnivores)			
Canidae	281 (2.5%)	U.S. zoos	[90]
*Panthera* Felidae	42/122 (34.4%)	North American zoos	[43]
	2/80 (2.5%)	North and South American zoos	[91]
		European zoos	
	5/38 (13%)	North American zoos	[92]
*Non-Panthera* felids	10/97 (10.3%)	North American zoos	[43]
	1/115 (0.9%)	North and South American zoos	[91]

* For cases/total population assessed, see the referenced literature. ** Evaluated necropsies 1988–2019 [16]. *** Evaluated necropsies from 1975 to 1995 at National Zoo [32].

**Table 3:** Prevalence of uterine leiomyomas among animals reported to the Exotic Species Cancer Research Alliance (ESCRA) database (animals with neoplasia).

Species	Uterine Leiomyoma Cases/Total Cases of Cancer in ESCRA Database (Prevalence)
African lion * (*Panthera leo*)	6/33 (18%)
Bison (*Bison bison*)	2/2 (100%)
Black lemur (*Eulemur macaco*)	3/4 (75%)
Coquerel’s giant mouse lemur (*Mirza coquereli)*	2/6 (33%)
Capybara (*Hydrochoerus hydrochaeris*)	1/4 (25%)
Caracal (*Caracal caracal*)	1/1 (100%)
Chimpanzee ** (*Pan troglodytes*)	2/4 (50%)
Chinchilla (*Chinchilla chinchilla*)	1/2 (50%)
Cottonmouth (*Agkistrodon piscivorus*)	1/4 (25%)
Giant anteater (*Myrmecophaga tridactyla*)	1/1 (100%)
Greater Rhea (*Rhea americana*)	1/4 (25%)
Mandrill (*Mandrillus sphinx*)	1/1 (100%)
Nile lechwe (*Kobus megaceros*)	1/1 (100%)
Patas monkey (*Erythrocebus patas*)	1/3 (33%)
Pygmy goat (*Capra hircus*)	1/3 (33%)
Pygmy hippopotamus (*Choeropsis liberiensis*)	1/1 (100%)
Red river hog (*Potamochoerus porcus*)	1/1(100%)
Red wolf (*Canis rufus*)	1/3(33%)
Skunk (*Mephitis mephitis*)	1/3 (33%)
Slender tailed meerkat (*Suricata suricatta*)	2/5 (40%)
Tufted grey langur (*Semnopithecus priam*)	1/2 (50%)
Zebra, multiple subspecies (*Equus* ssp.)	1/71 (1.4%)

* African lion with leiomyoma that was diagnosed and treated prior to death: surgical removal through ovariohysterectomy. ** Chimpanzee with leiomyoma was assessed for surgery, but surgery was not possible due to size and location of tumor.

## 5. Reproductive Consequences of Tumors in Humans and Fertility Preservation Approaches

The impact of tumors on reproduction are best documented in humans, and fertility preserving treatment approaches have been established. Many tumor types, both benign and malignant, are reported to negatively impact reproduction in humans, including reproductive tumors: uterine, ovarian, vaginal, cervical, vulvar, mammary, testicular, penile, and prostate neoplasias [93]; and non-reproductive tumors: thyroid, pituitary and adrenal neoplasms, and any tumor type requiring treatment that destroys reproductive or gonadotropin-producing organs [94,95,96,97,98]. Negative reproductive impacts include physical and endocrinological effects of tumors as well as surgical and toxic effects of treatment [99]. Unsurprisingly, tumors in the reproductive tract have a high potential to affect fertility [100]. Less obvious, tumors outside of the reproductive tract can also affect reproduction by growing large enough to press on and obstruct the reproductive organs, physically invade the anatomic region, or affect reproductive endocrine function [22]. Humans have a long post-reproductive life [101]; therefore, age at tumor diagnosis is a factor that affects the reproductive impact of the tumor. The average age of menopause is 51 years, and in western, industrialized societies, most women do not reproduce after age 40 [102]; therefore, only tumors that develop prior to the fifth decade of life impact female reproduction.

Similar to elephants, uterine leiomyomas (fibroids) are one of the most commonly diagnosed tumors in humans, and they affect fertility and pregnancy. Depending on size, location, and number, fibroids can impair sperm transport, block fallopian tubes, distort the endometrial lining, reduce implantation rates, increase risk of pregnancy loss, and lead to complications during delivery [103]. By age 50, the estimated cumulative incidence of leiomyomas in women is 70 to 80%, and reproductive issues may occur in women who develop uterine lesions at a young age [104,105]. Shared risk factors for uterine fibroids in humans and other mammals include increasing age and nulliparity [48,106]. Additional risk factors for these lesions in humans include early menarche, late menopause, obesity, high gonadotropins, ethnicity, and genetics (heritability) [107]. A genome-wide association study (GWAS) with data from 16,595 uterine leiomyoma affected women and 523,330 unaffected control individuals linked leiomyoma development with variants in genes involved in tumorigenesis (*TP53*, *TERT*, *ATM*, and *OBFC1*) and genes involved in hormone metabolism (*CDC42*/*WNT4*, *GREB1*, *MCM8*, and *SYN1*/*ESR1*) [51].

In women, uterine fibroids are only treated when they cause symptoms such as bleeding, pain, and infertility. Contraceptive treatment is one approach used to suppress the development and progression of uterine fibroids [108]. A number of other approaches can be used, including surgery (myomectomy) and hormone therapy [109]. Hormone therapy includes GnRH analogues, progestins, progestin and estrogen combinations, and ulipristal acetate (the morning-after pill) [110].

Diagnosis of cancer prior to puberty in humans often has detrimental effects on future reproductive success. Because infertility is one of the most common morbidities associated with pediatric cancer [111], multiple treatments to preserve fertility in children with cancer have been developed [112,113]. These approaches can be considered for feasibility in other animals with uterine leiomyomas and other types of tumors. Pediatric cancer is the third leading cause of death in children [114]. Improved treatment has increased survival to 90% for some childhood cancers, such as acute lymphoblastic leukemia, but survival still remains much lower for other specific childhood tumors [115]. An individual must survive past puberty to successfully reproduce, and therefore pediatric cancers with poor survivability negatively impact reproductive success. However, even individuals who survive pediatric cancer often face reproductive challenges, due to the toxic effects of treatment on the reproductive organs before reproductive years [111,116]. Adults diagnosed with malignant tumors, including reproductive neoplasias, can also experience the negative reproductive effects of treatment, depending on their age and reproductive stage at time of treatment. The treatments used to kill tumor cells (chemotherapy and radiation) also kill germ cells, which can lead to sterilization [111]. For children that need toxic, yet lifesaving, treatments prior to puberty, experimental approaches of ovarian and testicular cryopreservation have led to successful conception and birth in a small number of patients who had ovarian tissue reimplanted after treatment. More options are available for post-pubertal children and young adults requiring treatment including sperm and oocyte preservation. These approaches, which are no longer considered experimental in humans, have resulted in many births. Another option for adults with a partner is embryo cryopreservation, a well-established fertility preservation approach in humans. Both oocyte preservation and embryo cryopreservation require time to stimulate ovaries prior to harvesting mature oocytes. Unfortunately, in some cases, these may not be viable options since treatment cannot be delayed due to the patient’s condition and cancer progression at diagnosis [117]. One additional approach to preserve fertility that can be used in post-pubertal children and women is ovarian suppression with gonadotropin-releasing hormone (GnHR) agonists. However, experts debate the efficacy of this approach, and it is only recommended when more established fertility preservation techniques are not an option, such as when time needed to stimulate the ovaries for the more established techniques is not recommended [112,113]. These approaches, both established and experimental, can be considered for feasibility to preserve fertility across species.

## 6. Potential Impact of Reproductive Tumors and Interventions to Preserve Fertility in Elephants

Knowledge related to the impact and various treatment approaches to preserve fertility in humans can be considered when reviewing and researching the impact of tumors and treatment approaches in elephants. The majority of neoplasias in elephants under human care are diagnosed post mortem [16]. Because most of these elephants are elderly, the true impact of neoplasia on elephant reproduction is unclear. While it is well documented that a high percentage of Asian elephants develop uterine leiomyoma [7,15,16], the age of onset is unknown. A 15-year-old Asian elephant under human care in Thailand was euthanized due to prolonged weakness, recumbency, and lack of response to treatment. At necropsy, the elephant was diagnosed with multiple uterine leiomyomas, 5–10 cm in diameter [35]. This highlights an unmet need to use transrectal ultrasonography to enable screening of elephants during their reproductive years to understand the average age of onset for these reproductive lesions. If reproductive tumors develop frequently in young elephants, then the overall impact on reproduction is higher than if they develop in older elephants with fewer opportunities to reproduce. At the same time, free-ranging elephants have a short post-reproductive lifespan compared to humans and their interbirth interval can actually decrease as they age [11,12], which means that the lesions could still have a significant impact when diagnosed in older elephants. About 30% of African elephant females will live longer than the age at which fertility declines (49 yr) [11]. While the average post-reproductive life of these females is 11–17 years, many females continue to reproduce alongside their daughters, well into older age [12]. After reproductive decline, grandmothers can further augment their own inclusive fitness by providing knowledge, and protecting daughters’ offspring [11]. The age-specific rate of reproduction, with calves surviving to 12 months, was higher for daughters with a surviving mother and increased with longer reproductive overlap between mother–daughter pairs [11]. While the authors did not offer an evolutionary explanation for this benefit, older females are a repository of knowledge (e.g., where to find water and food sources) that can help daughters raise offspring longer than 12 months.

The prevalence of uterine leiomyoma and other types of neoplasia is unknown in free-ranging elephants. While associated with older, nulliparous elephants [7], the high prevalence of uterine leiomyoma in Asian elephants under human care [16] suggests that these tumors may also develop in free-ranging animals. Reproduction can be delayed in the wild during conditions of limited resources and other unfavorable environmental conditions [14,118], increasing the chances that these lesions affect free-ranging elephants. Future studies need to confirm the potential impact of these lesions and their association with age and parity in free-ranging elephants, but the feasibility of these studies is questionable. Necropsy of free-ranging elephants remains challenging due to decomposition and predation of bodies prior to discovery, as well as access to and costs of histopathology [119].

Uterine leiomyomas in humans and other animals are known to negatively impact reproduction [64,103], and some evidence that these lesions may negatively impact elephant reproduction already exists [7]. For example, a 24-year-old, multiparous Asian elephant was diagnosed with uterine leiomyoma in the submucosa of the left uterine horn after pregnancy loss and reabsorption at 18 weeks of gestation [39]. It is not possible to prove that pregnancy loss was related to the tumor, but it also cannot be ruled out as a factor. Asian elephants under human care undergoing fertility monitoring have also been observed with uterine lesions by ultrasound (unpublished observation by co-authors D. Schmitt and W.K. Kiso). This observation, combined with multiple cases of young (12-, 13-, 15- and 21-year-old) Asian elephants with uterine leiomyomas [16,32,33,35] (Table 1), suggests that at least a percentage of elephants develop these lesions at ages young enough to impact reproduction. Future studies to document the presence of these lesions in young females and reproductive outcomes will help to determine if an association exists. Detailed records of size and location of lesions that negatively affect conception and pregnancy can guide future interventions to improve outcomes.

Fertility preservation techniques developed for humans with leiomyomas [107] could be explored for elephants; however, these approaches may not be technically feasible for elephants. For example, the most common treatment for women with large uterine leiomyomas is surgical removal [107]. Surgical removal of these lesions in elephants would require abdominal surgery, which has been unsuccessful in adult elephants [120,121,122]. Surgical incisions and wounds in elephants are highly susceptible to infection and dehiscence (incision separation following surgery) [122], and surgical removal of internal tumors is currently not feasible.

A high prevalence of uterine leiomyoma in Asian elephants compared to no known reports in African elephants suggests that genetics contribute to risk. In this case, genetic differences between the two species likely result in increased risk in Asian elephants. However, alterations in genes within a species can increase an individual’s risk compared to the population as a whole. For example, genetic alterations associated with a predisposition for uterine leiomyoma development were identified within humans and rats [51,123]. An analysis of Asian elephant genomes may reveal alterations in these same genes that increase leiomyoma risk in Asian elephants and other animals. Further genome-wide analysis comparing Asian elephants that do not develop uterine leiomyoma to those that do may identify additional, within species genetic alterations associated with these tumors. Identification of genetic alterations that increase risk of uterine leiomyoma can enable earlier screenings and interventions for elephants at high risk, if genetic screening protocols are developed.

Because nulliparity and increasing age are associated with uterine leiomyomas [7], initiating reproduction (natural or assisted) as soon as females begin cycling and reach an adequate size to safely mate and give birth may help to suppress these lesions in the long-term. Adding routine ultrasound examinations when monitoring and preparing females for reproduction may be beneficial to identify lesions that may interfere with conception, pregnancy, or birth [32]. In humans, pregnancy is often delayed with contraception, and low dose estrogen plus progesterone contraceptive use is thought to be protective against fibroid development [124]. In cases where elephant reproduction needs to be delayed, reversible contraceptives could also be beneficial to suppress these lesions. Unfortunately, this approach is also currently not feasible for elephants. When an estrogen-only contraceptive patch similar to the human contraceptive “pill” was tried in elephants, the females went into a state of continuous false estrus and were harassed by bull elephants [125,126]. However, based on the effect in human women, estrogen alone would not be beneficial to prevent or reduce the size of uterine leiomyomas. The feasibility of using a contraceptive with low dose estrogen plus progesterone as a treatment or preventative for leiomyomas without inducing false estrus in elephants is unclear.

Other contraceptive approaches have been used successfully in elephants, including porcine zona pellucida (pZP) and gonadotropin releasing hormone (GnRH) vaccines [7]. pZP is harvested from pig ovaries and administered as a vaccine. The vaccine stimulates the production of antibodies against pZP that block oocyte sperm receptors. Reports of the effects of pZP on ovarian activity vary, and the cause of this variability is difficult to ascertain as the species, duration, and formulation used in the studies varies. However, while some formulations of pZP do induce ovarian suppression in mares after some time [127], others seem to still allow mares to cycle [128]. The use of pZP had no effect on folliculogenesis of white-tailed deer [129]. In African elephants it has been documented that at least some females will continue to cycle while treated with pZP [130,131]. Leiomyomas will not be suppressed in females that do cycle, but may in females that cease folliculogenesis. Therefore, GnRH vaccines may be a better choice when the goal is to suppress ovarian activity [132], because GnRH vaccines reduce gonadal hormone levels, including luteinizing hormone [7]. In some cases (Asian and African elephants), GnRH vaccines prevented cycling and even decreased or resolved uterine leiomyomas [132,133,134]. However, in other cases (African elephant) GnRH vaccine did not induce anestrus [135]. GnRH vaccines have not been in use long enough to document reliable reversibility in non-domestic species after prolonged use. Additionally, because the source of this treatment (produced by different companies) and dosing has been inconsistent across studies [7], the efficacy of this treatment needs to be assessed with controlled dosing across study groups. Of course, to be useful for fertility preservation, reversibility of cycling suppression is essential. Results to date show reversibility in some cases, but needs further investigation, especially if females are to be treated long-term [7]. Controlled studies that measure the effects and reversibility of different doses of GnRH vaccines are needed, but again, the feasibility of these types of studies in elephants is questionable. Controlled studies are possible, however, as they were performed to study the efficacy of pZP in free-ranging elephants in parks and game reserves in South Africa [136].

As discussed, oocyte harvesting is used successfully in children and young adults diagnosed with cancer prior to initiating treatments that cause sterility [112]. Oocyte harvesting could be considered for elephants, but this approach has shown limited success in non-domestic species [62]. In felids, there has been some success with freezing fertilized embryos [137], and rhinoceros embryos have been generated through in vitro fertilization [138]. While some assisted reproductive techniques are used in elephants [7], the authors are unaware of any attempts to perform in vitro fertilization to produce and freeze elephant embryos. Differences in the anatomy of elephants compared to other animals may present challenges for oocyte retrieval, but if successful it could be beneficial for conservation.

The generation of induced pluripotent stem cells (iPSCs) also holds promise for the conservation of endangered species [139]. Skin fibroblasts from the functionally extinct northern white rhinoceros were reprogrammed into iPSC, and the iPSC were differentiated into primordial germ cells, which are the precursors to oocytes and sperm [140]. This study suggests that in the future the northern white rhino could be saved from extinction by generating embryos with germ cells differentiated from iPSC. If iPSC can be generated from elephant cells, then it will be a great approach to preserve genetic material and potentially protect these animals from extinction. However, to the authors knowledge, elephant iPSCs have not yet been successfully generated. It is unknown why elephant cells resist reprogramming, but it may be related to the activity of a tumor suppressor gene called *TP53* [141,142,143]. If the issue can be solved, then this approach perhaps could be the most promising. Primary elephant skin fibroblasts grow well in culture and cells from several elephants are available for reprogramming attempts [142,144,145,146].

## 7. Conclusions

The prevalence of both benign and malignant neoplasia differs between African and Asian elephants, with Asian elephants more frequently diagnosed and negatively affected by both. Malignant tumors are not frequently diagnosed in either species, potentially due to strong defense mechanisms that protect against malignant tumors, [15,142,143,147]. Benign uterine tumors (including leiomyomas) are frequently diagnosed in Asian elephants [15,16], and their negative impact on fertility is already documented in elephants and other species [18,19,39,64]. The frequency and impact of neoplasia in elephants needs further investigation.

Additionally, the reproductive impact of various treatment options for elephants with malignant tumors should be considered along with fertility preservation techniques prior to treatment. Preventative and treatment options are available and have been used in other species; however, more studies are needed to assess their efficacy and impact in elephants. Future studies that document reproductive outcomes in elephants with benign and malignant tumors, as well as with tumor treatment approaches are needed to develop best practice approaches that can increase reproductive output.

Until those studies can be completed, the existing literature suggests that opportunities exist to minimize female reproductive lesions to improve general health and to enhance reproductive outcomes. While each elephant and/or facility may have unique circumstances, recommendations supported by this review are: (i) start breeding females young (natural breeding or artificial insemination; with safety considerations of social compatibility and/or physical size); (ii) keep inter-calf interval as low as possible (i.e., average of 4–5 years inter-calving interval); (iii) perform routine transrectal ultrasonography from an early age to monitor development and progression of reproductive lesions; and iv) suppress ovarian hormones (i.e., GnRH vaccination or synthetic progestin: altrenogest) in females with reproductive lesion progression and in those that will no longer breed (post-reproductive or for management reasons).

Every newborn elephant is important for species survival, and a better understanding of the impact of tumors on elephant reproduction is urgently needed to ensure successful conception and complication-free pregnancy and birth in elephants.

## Figures and Tables

**Table 1:** Reproductive lesions reported in female elephants.

Diagnosis	Elephant Species	Age (Years)	Reported Cases/Total (Prevalence)	Reference
Uterine leiomyoma	*E. maximus*	12–57	19/27 (70%)	[33]
		38 ± 8.49 (mean ± SD for acyclic)	6/37 (16%)	[34]
		NR	27/27 (100%)	[38]
		13–71	57/80 (71%)	[16]
		52	1	[39]
		21–46	20/56 (36%)	[32]
		15 & 50	2	[35]
		50	1	[36]
		58	1	[37]
		39–59 (3/7 reported)	7/19 (37%)	[15]
Uterine adenocarcinoma	*E. maximus*	45–65	8/80 (10%)	[16]
		56	1	[40]
		59 (1/3 reported)	3/19 (16%)	[15]
Anaplastic carcinoma (uterus)	*E. maximus*	60	1/80 (1%)	[16]
Carcinoma in situ in endometrial polyp	*E. maximus*	57	1/80 (1%)	[16]
Peripheral neuroectodermal tumor (uterus)	*E. maximus*	48	1/80 (1%)	[16]
Angiosarcoma (uterus)	*E. maximus*	50	1/80 (1%)	[16]
Anaplastic sarcoma (pelvic mass of presumed uterine origin)	*E. maximus*	53	1/80 (1%)	[16]
Endometrial cysts	*L. africana*	NR	NR	[7]
	*E. maximus*	NR	NR	[7]
Ovarian carcinoma	*E. maximus*	NR	1/80 (1%)	[16]
Ovarian cysts	*L. africana*	NR	NR	[7]
	*E. maximus*	NR	NR	[7]
	*E. maximus*	NR	11/80 (14%)	[16]
Bilateral multilocular serous ovarian cystadenoma	*L. africana*	59	1	[41]
Hyperplastic endometrial disease	*L. africana*	12–46	2/13 (15%)	[33]
	*E. maximus*	12–57	18/27 (67%)	
Vestibular cysts	*L. africana*	NR	NR	[31]
	*E. maximus*	NR	NR	
Vestibular polyps	*L. africana*	NR	NR	[31]
Vaginal leiomyoma	*E. maximus*	NR	1	[16]
Hyperplastic, polyploidy or papillomatous mucosal lesions of vagina/vulva	*E. maximus*	NR	10/80 (13%)	[16]
Vagina polyps	*L. africana*	28	3/19 (16%)	[15]
	*E. maximus*	30 & 40		
Vulvar polyps	*E. maximus*	45	1/19 (5%)	[15]
Uterine polyps	*E. maximus*	50	1/19 (5%)	[15]
Uterus undifferentiated malignant neoplasm	*E. maximus*	NR	1/19 (5%)	[15]

NR = not reported.

## Data Availability

Deidentified data are available through an approved research request to the Exotic Species Cancer Research Alliance (https://escra.cvm.ncsu.edu/, accessed on 7 June 2022).
